# COVID-19 as an opportunity for minimally-invasive dentistry: a national cross-sectional survey

**DOI:** 10.1186/s12903-022-02432-7

**Published:** 2022-09-12

**Authors:** Ilze Maldupa, Olga Slepcova, Ilona Vidulskane, Anda Brinkmane, Egita Senakola, Sergio E. Uribe

**Affiliations:** 1 Department of Conservative Dentistry and Oral Health, Riga Stradins University, Dzirciema iela 20, Riga, 1007 Latvia; 2 School of Dentistry, Universidad Austral de Chile, Valdivia, Chile; 3 Baltic Biomaterials Centre of Excellence, Headquarters at Riga Technical University, Riga, Latvia

**Keywords:** COVID-19, Dentistry, Survey research, Dental caries, Decision-making

## Abstract

**Background:**

During the COVID19 pandemic, the Latvian government issued first absolute restrictions (elective treatments prohibited, only emergency care) and later relative restrictions (preference for non-aerosol-generating procedures (AGP) and emergency care) on dental care. This study aims to assess the impact of these restrictions on the decision made by Latvian dentists about caries treatment.

**Methods:**

A Survey-based cross-sectional study was used. A minimum sample size of 174 dentists was estimated for national representativeness (N = 1524). The questionnaire was developed by experts and sent three times via email to Latvian dentists from July to September 2020 and was also delivered in printed form at two national conferences in September and October 2020. Descriptive statistics were calculated.

**Results:**

We received 373 completed questionnaires, with a total response rate of 24.5%. Under the recommendation to reduce AGP for the treatment of uncomplicated caries, 10% of the dentists stated that they would stop attending, 54% would only attend emergencies, and 36% would attend as usual. Under prohibition, the percentages are 15%, 74%, and 11%, respectively. Regarding the type of treatment, more than 75% would opt to proceed with selective caries removal for both primary and permanent teeth and 10% for extraction.

**Conclusion:**

Latvian dentists are willing to treat patients with caries during the pandemic and state that they prefer to use non- or minimally invasive and less aerosol-generating methods for caries treatment.

**Supplementary Information:**

The online version contains supplementary material available at 10.1186/s12903-022-02432-7.

## Background

Most intraoral dental procedures generate aerosol [1], particularly those related to dental caries treatment [2], the main pathology affecting people around the world [3]. Since the appearance of COVID-19, the issue of aerosol-generating procedures (AGP) has gained importance because it could be a potential route of virus transmission. These AGPs could transmit bacterial particles or viral infections [4]. These infected aerosols can contaminate surfaces and objects, creating fomites or contaminated surfaces [5], even though their potential for contagion could be low on these surfaces [6]. Viable SARS-CoV-2 virus and RNA causing COVID-19 detected by RT-PCR can be found on these surfaces for periods ranging from hours to days, depending on the type of surface, temperature, and environmental humidity, particularly in healthcare facilities treating patients with COVID-19 [7], posing potential risks of infection transmission [8].

During the COVID19 pandemic, the choice of treatment by the dentist has become critical, as the route of transmission of the virus includes saliva and droplets generated during dental procedures [9]. Therefore, any intervention that minimizes the generation of aerosols may be preferable to the risk of contagion during dental care [8]. This has led authorities to provide recommendations on procedures to prevent infection during dental care [10] or even to restrict or prohibit elective dental care.

Following the declaration of the COVID-19 pandemic, the Latvian government ordered a ban on all elective dental care, and specifically any AGP, as of March 25, 2020 [11]. Subsequently, on May 13, 2020, it lifted the ban and issued a recommendation to delay elective dental treatments and prefer non-AGPs [12]. On the other hand, the high prevalence of filled teeth (Care index = 0.83) indicates the traditional invasive caries treatment approach is preferred by Latvian dentists [13], but there is no specific data on the subject. These national recommendations to restrict or prohibit some dental actions provide an opportunity to evaluate what dentists would do in cases of restricted dental care. For example, would they opt to replace AGPs with non-AGPs, attend only emergencies, or definitely not attend at all? This is particularly important because in general, less invasive, non-AGPs [2] have shown evidence of effectiveness [14], yet there are reports that they are underutilized [15]. Hence, the recommendations to prohibit AGPs or recommend that AGPs be avoided during the pandemic could therefore constitute an opportunity to use less invasive treatments against dental caries. The aim of this study is to assess the impact of these restrictions on the decision made by Latvian dentists about caries treatment.

## Methods

### Study design

Cross-sectional study. The information was collected by survey, so we followed the SURGE recommendations for reporting survey research [16].

### Ethical approval and privacy

Ethical approval for this study was obtained from the RSU Ethics Committee (Nr. 6-1/07/13, 2020) within the framework of the VPP-COVID-2020/1-0011 project and was conducted in accordance with the Declaration of Helsinki [17, 18]. The study sought to evaluate different aspects of dental practice during the COVID-19 pandemic. Participants' informed consent was included in the questionnaire, which indicated the nature of the research, the risks, and benefits, and that completing the questionnaire was considered acceptance to participate; hence, participants gave their consent by completing the questionnaire voluntarily and anonymously. Email addresses were unavailable to researchers since questionnaires were sent out by Latvian Dental Association (LDA) employees, who have access to email addresses with the users' consent. The online and printed questionnaire did not record any personal data that could identify the respondent.

### Research tool

We modified the questionnaire of Mejare et al. on dentists' diagnosis and treatment decisions about dental caries [19], adding two sets of questions about what they would do when faced with a recommendation to limit elective dental care or when faced with a ban on elective dental care and about what treatments for dental caries they would use in each of these scenarios. We wanted to find out whether Latvian dentists would choose to change their treatments or cancel them. The original Mejare et al. (1999) questionnaire was translated into Latvian by two translators independently and then back-translated into English. A group of academic dentists with expertise in both languages compared the translations and made changes until an equivalent Latvian version was obtained. For the section about the attitude toward COVID-19 constraints, we created 6 questions. The face-validity was established in two stages. First, we asked a group of experts to evaluate the questions. Then, we asked a survey expert to evaluate the construction of the questions to correct any confusing or double-barreled questions. We then applied a pilot test on 8 general dentists to identify problems and ensure correct questions interpretation. Internal consistency was evaluated using Cronbach's Alpha, establishing a minimum value of 0.7. Since all the questions obtained values equal to or greater than 0.7, they were retained. Reliability was evaluated by means of a test–retest two weeks after the first application, and we calculated the Pearson correlation coefficient, leaving all items with a value greater than 0.7. Reliability showed a weighted score for all items of 0.78, which is considered good [20]. No items were eliminated. The details of the calculations can be found in the data repository [21].

The final instrument comprises 10 questions included in the final version of the questionnaire (available as Additional file 1) about uncomplicated caries treatment, i.e., caries with reversible pulpitis. The questions were:

1. Demographic data.
2. What dentists would do if AGPs were banned or AGPs were recommended to be avoided.
3. What treatment they would use, for primary or permanent teeth, if they had to treat a patient with caries if AGP treatments were prohibited or recommended to be avoided.

### Population target and sample selection

The target population was practicing dentists in Latvia. All practicing dentists are registered with the LDA, and as of March 2020, there were 1524, of whom 1092 had consented to receive news and information of interest in their email. Additionally, to include those who had not consented to receive information in their mail or who did not regularly use their mail, printed copies were hand-delivered at a national LDA conference (September 16–17, 2020) and three local courses in Riga, the capital of Latvia in October 2020. We assumed that 15% of dentists would prefer non-invasive methods based on expert estimation, and assuming a 5% margin of error, we calculated a minimum sample size of 174 respondents to estimate the true proportion with a 95% confidence interval [22].

### Survey administration

The survey was sent electronically to the 1092 registered LDA dentists who consented to receive information by email. We sent out via email an invitation to fill in the online questionnaire 3 times. Each online questionnaire had a digital token to prevent it from being completed more than once. Each questionnaire had to be with all items answered in order to be sent. Questionnaires were also printed in paper (n = 294) format to be offered to dentists by some of the authors in face-to-face lectures in order to reach the dentists who do not use email or do not want to be sent information via email. The exclusion criteria were an incomplete or invalid questionnaire. No incentives were offered.

### Analysis

The data from the printed questionnaires were entered into the online version of the questionnaire. The data from the online questionnaire were then exported to a csv file for statistical analysis. Dentists were classified according to their responses as traditional or minimally invasive. Using questions from Mejare et al., the profile of dentists was assessed in two categories: minimally invasive if the ordinal values of the four questions did not exceed 4 and traditional or restorative approach dentists if the sum of the scores was 5 or more.

Because of the COVID-19 pandemic, it was hypothesized that there might be situations where aerosol-generating methods might be banned or recommended not to be used. Based on these descriptions, dentists chose which of the proposed methods they would be willing to use. Responses were coded according to the method's compliance with the principles of minimally invasive dentistry. The lower the score, the more likely the dentist would choose minimally invasive methods to treat uncomplicated caries. The experts chose the reference score as the sum of the responses for which dentists would likely use non-invasive and minimally invasive techniques but unlikely to use invasive techniques. This sum is 24 in the hypothetical case of a recommendation not to use aerosol-generating techniques and 20 in the case of a ban. So, dentists with a score of 20/24 or less were classified as minimally invasive and those with a score of more than 20 or 24 as traditional, restorative approach. We evaluated differences in the responses using a 2-sample test for equality of proportions with continuity correction. Statistical differences for Likert responses were analyzed using McNemar's test for the variable of what type of care (would not attend, emergencies only, normal) and the proportion test to compare the different treatment recommendations during the recommendation and prohibition periods. The difference in change between the ratio at baseline and during the prohibition or recommendation period was explored with the chi-square test. To explore some variables that could explain a shift towards more or less invasive decisions, changes were coded as more or less invasive, and a generalized linear regression test was applied with the independent variables sex, year of graduation, and whether or not he/she is a specialist, and according to the type of patients he/she claims to see. The statistical significance level was set at 5%. All statistical analyses were performed with R [23] and the Tidyverse [24], janitor [25], gtsummary [26], sf [27] and sjPlot [28] packages.

## Results

We received 373 completed questionnaires, with a total response rate of 24.5%. Of the 1092 questionnaires submitted online, we received 235 questionnaires, response rate = 21.5%. Of the 294 questionnaires submitted on paper, we received 138 completed questionnaires, response rate = 46.9%. The demographic characteristics of the respondents are shown in Table 1.

**Table 1:** Demographic characteristics of the respondents

Characteristic	N = 373^a^
*Sex*	
Female	344 (92%)
Male	29 (7.8%)
Specialist degree	29 (7.8%)
Years since graduation	22.2 (0, 47)
*Type of patients served*	
Only children	69 (18%)
Children and adults	223 (60%)
Only adults	81 (22%)

^a^Statistics presented: n (%); mean (min, max)

**Fig. 1:**
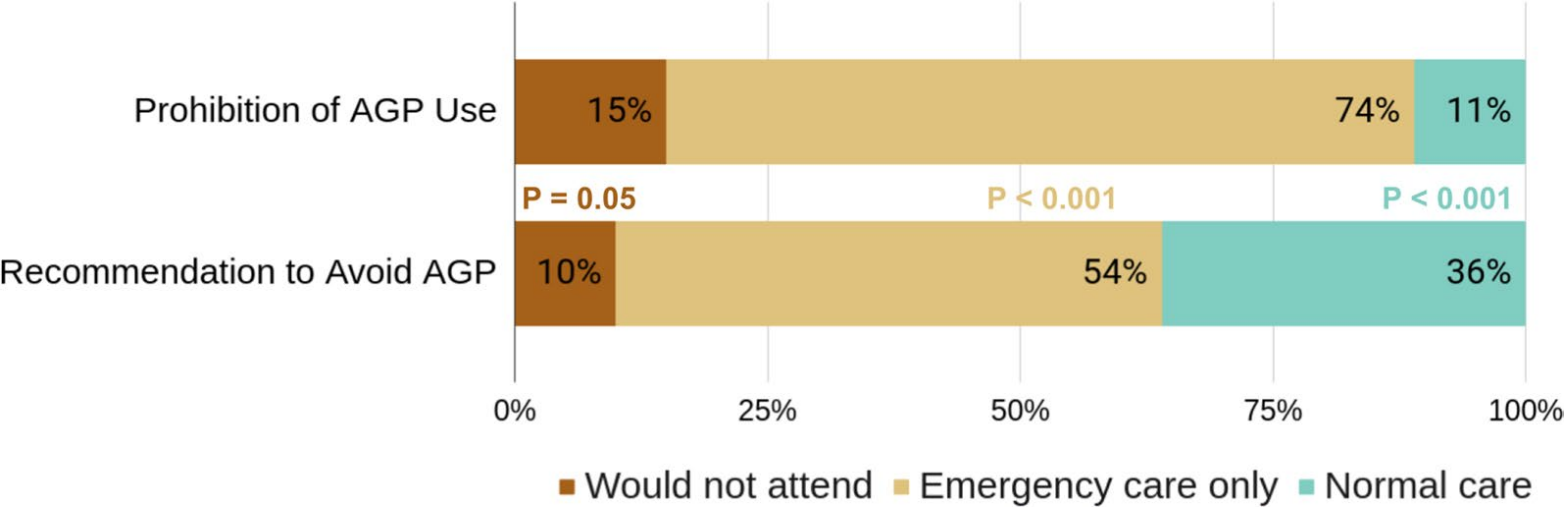
Dentists’ choice to admit patients during the Covid-19 epidemic, depending on the existing ban or recommendations not to use AGPs

The proportion of dentists who stated that they would not attend under prohibition or recommendation of PGA had differences that were not significant (15–10%), but we did detect differences in dentists who would do only emergency procedures and normal care under prohibition or recommendation to avoid PGA, as seen in Fig. 1.

**Fig. 2:**
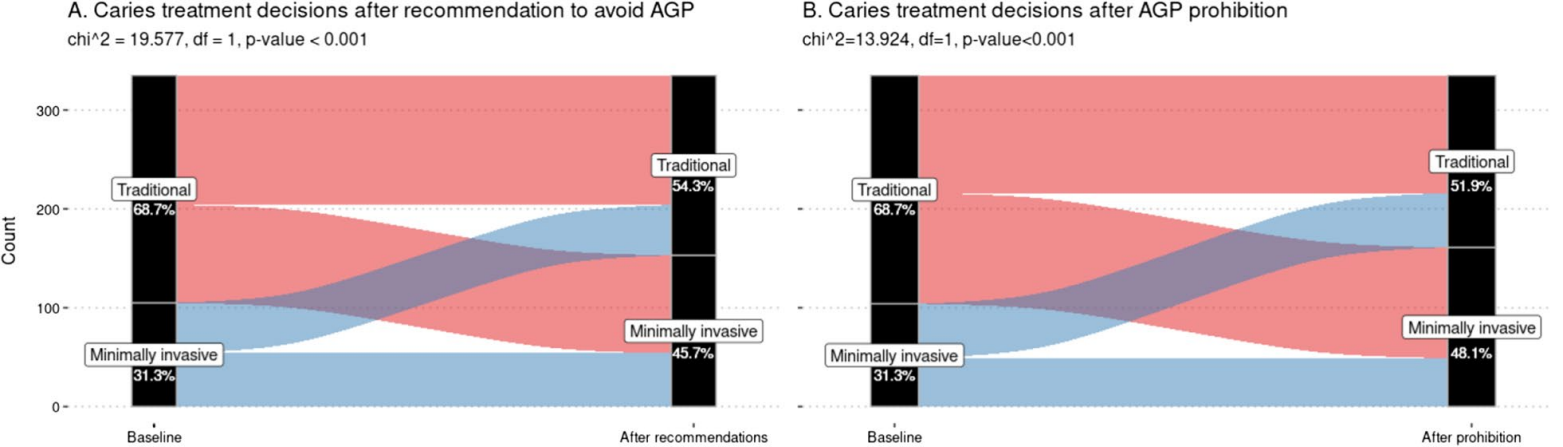
The proportion of change in treatment decisions by dentists for the situation in which they recommend avoiding PFAs or prohibiting them. The differences in proportions vary significantly in both conditions with respect to the baseline situation

The proportion of change in the classification of dentists according to whether they were classified as traditional or minimally invasive is shown in Fig. 2. The percentage of participants who report opting for conventional or invasive treatments differs significantly after the recommendation to avoid AGP or ban AGP compared to baseline.

**Fig. 3:**
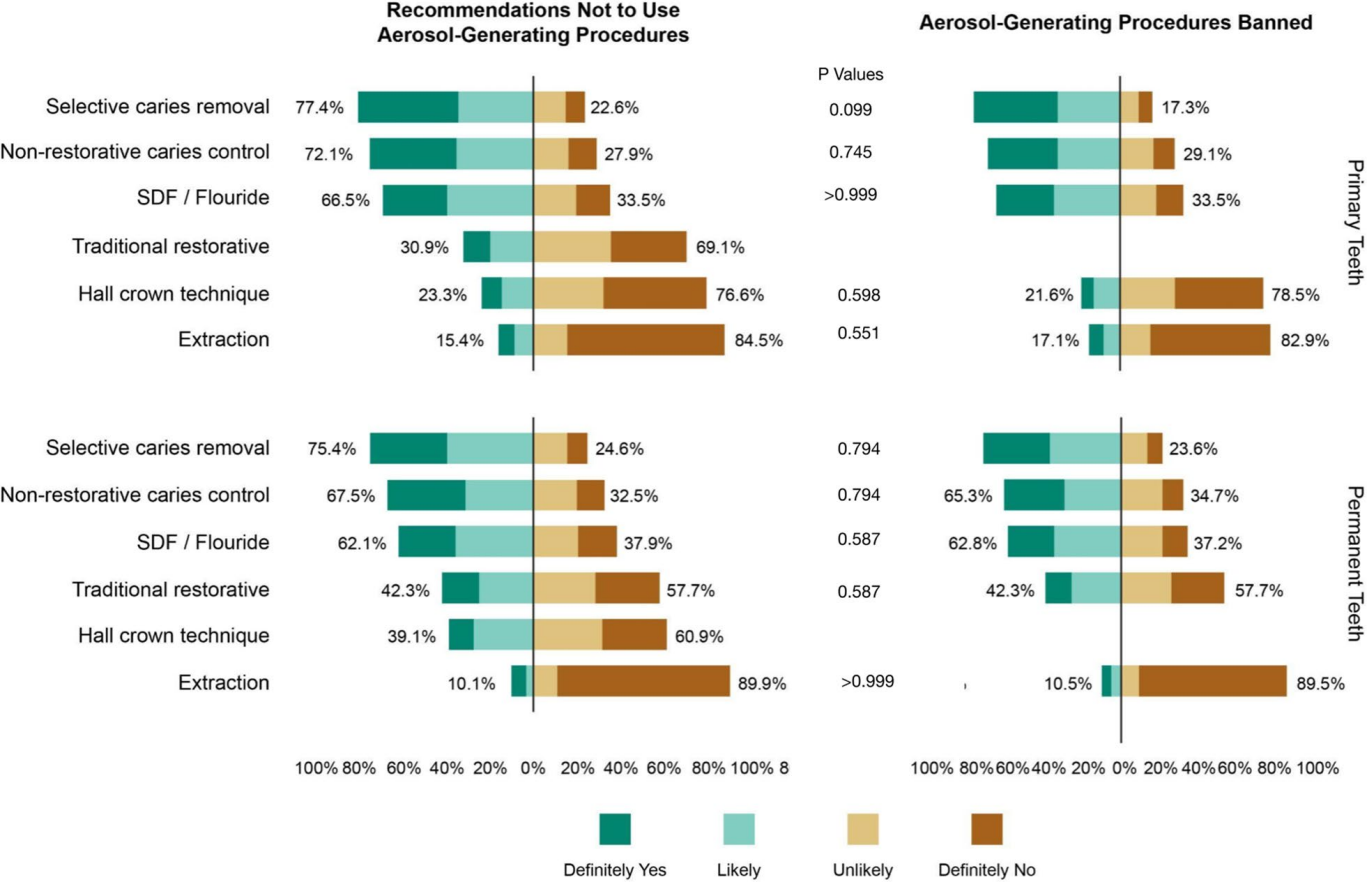
Latvian dentists’ choice of uncomplicated caries treatment methods depends on the existing restrictions due to the Covid-19 pandemic

Regarding the type of treatment that would be performed in a case of uncomplicated caries in the event of a recommendation to avoid AGP or prohibition for both primary and permanent teeth, more than 75% would opt to proceed with selective caries removal. The procedure that the highest proportion of dentists reported avoiding in both cases and for both dentitions was extraction. Regarding the type of treatment that would be performed in a case of uncomplicated caries in the event of a recommendation to avoid AGP or prohibition for both primary and permanent teeth, more than 75% would opt to proceed with selective caries removal. The procedure that the highest proportion of dentists reported avoiding in both cases and for both dentitions was extraction. The details are shown in Fig. 3. We found no significant differences between the proportions of responses for the period of recommendation to avoid or ban AGP.

We fitted a logistic model (estimated using a generalized linear model) to predict change to minimally invasive decisions during the AGP ban or recommendation to avoid AGP with sex, graduation year, specialist, and type of patients. The model's explanatory power is weak (Tjur's R2 = 0.02). The model's intercept, corresponding to Sex = Female, Graduation Year = 1973, Specialist = Yes and Patients = Adults and children equally, is at − 8.01 (95% CI [− 44.97, 28.91], p = 0.670). Within this model:

The effect of Sex [Male] is non-significantly negative (beta = − 1.10, 95% CI [− 2.57, − 2.66e−03], p = 0.081; Std. beta = − 1.10, 95% CI [− 2.57, − 2.66e−03]); the effect of Graduation_Year is non-significantly positive (beta = 3.44e−03, 95% CI [− 0.02, 0.02], p = 0.715; Std. beta = 0.05, 95% CI [− 0.20, 0.29]); the effect of Specialist [No] is non-significantly positive (beta = 0.34, 95% CI [− 0.55, 1.37], p = 0.480; Std. beta = 0.34, 95% CI [− 0.55, 1.37]); the effect of Patients [Small children very rare] is non-significantly negative (beta = − 0.66, 95% CI [− 1.42, 0.03], p = 0.072; Std. beta = − 0.66, 95% CI [− 1.42, 0.03]) and the effect of Patients [Only adults] is non-significantly negative (beta = − 0.08, 95% CI [− 0.70, 0.51], p = 0.785; Std. beta = − 0.08, 95% CI [− 0.70, 0.51]). The results for the analysis of factors that would explain the change of decision to more invasive interventions are similar and are shown in Table 2.

**Table 2:** Regression analysis of potential variables that could explain the shift toward more or less invasive treatment decisions

Characteristic	Less invasive			More invasive		
	OR	95% CI	p-value	OR	95% CI	p-value
Gender						
Female	–	–		–	–	
Male	0.33	0.08, 1.00	0.08	0.39	0.06, 1.41	0.20
Graduation year	1	0.99, 1.02	0.70	1.02	1.00, 1.05	0.09
Specialist degree						
Yes	–	–		–	–	
No	1.4	0.58, 3.94	0.52	2.18	0.61, 13.9	0.34
Type of patients						
Adults and children equally	–	–		–	–	
Small children very rare	0.52	0.24, 1.03	0.07	1.42	0.63, 3.00	0.41
Only adults	0.92	0.50, 1.66	0.87	0.85	0.34, 1.93	0.73

*OR* Odds Ratio, *CI* Confidence Interval

## Discussion

The COVID-19 pandemic has augmented mortality and morbidity both directly and indirectly, given disruption to medical and dental health systems functioning. Dentists have been faced with the decision to continue their care with the potential risk to their health and that of their team or to discontinue care [29]. In this study, we wanted to explore whether there were any positive aspects of the effect of this pandemic on dentistry. We found that most dentists in Latvia would continue to care for their patients' needs. We also found that most are willing to adopt less invasive treatments for the management of dental caries.

The dental profession should explore ways to return to routine patient care but do so safely for both patients and dental personnel. Dental personnel is among those most at risk of exposure to COVID-19 [30] according to an analysis of the physical aspects of each job, e.g., proximity to the patient. On the one hand, teledentistry provides protection by physically separating healthcare personnel from patients. But in the event that care is required, additional strategies are needed. Some strategies include the use of additional protective measures such as special suction equipment, FF2/FPP3 masks, and other additional protection measures [31], as well as adequate ventilation of the clinic [32]. On the other hand, there is an opportunity to incorporate treatments with evidence of effectiveness.

There is evidence from clinical studies showing that caries removal methods with low aerosol generation potential, such as Carisolv, Brix 3000, PapaCaries, and different types of lasers, are effective in removing caries-affected tissues, with restorations that have the same performance as those placed in conventionally treated teeth and with better patient acceptance [33]. Likewise, there is quality and consensus evidence showing that caries can be treated and intervened by non-operative and minimally invasive interventions in primary teeth [34], permanent teeth [35], and root surfaces in older adults [36]. Moreover, non-invasive and minimally invasive interventions generate less aerosol than traditional interventions [37]. However, these minimally invasive treatments are being underutilized [14]. For example, less than half of dental students in France consider sealants a routine treatment [38]. Likewise, for caries management, almost half of the dentists reject procedures with evidence of effectiveness, such as partial caries removal, according to the results of a systematic review [39]. A systematic review of dentists' reasons for using or not using more preventive approaches to the treatment of dental caries found that the main reason was economic [40]. The latest Latvian national epidemiological study shows that the prevalence of dental caries at age 12 is 98.5% [13]. The same study also finds that only 6.6% of 12-year-olds have sealants [13]. This shows that non-invasive treatments are rarely used in Latvia.

We performed a regression analysis to explore some variables that could explain the shift towards more or less invasive decisions without finding any that could independently explain the changes. This result suggests that the COVID constraints themselves could be the factor explaining the shift. It should be noted that this result should be considered only preliminary, and we hope that other groups can replicate it in other countries and carry out future hypothesis-driven rather than descriptive studies such as the present one.

Experts worldwide discuss why dentists are so slow to adapt to changes even when strong evidence of new approaches exists [41]. Our results suggest that COVID-19 is an opportunity to introduce non- or minimally-invasive treatments into clinical practice. The fact that most dentists in Latvia are willing to use non- or minimally invasive treatments for the treatment of dental caries is a positive result in the emergency situation represented by the COVID-19 pandemic. This represents an opportunity for positive change in the profession and joins other positive results, such as the confirmation of the correct management of infection control and the increased use of teledentistry by dentists. Thus, a study in the USA showed that fewer than 1% of dentists had tested positive for COVID-19 and that virtually all dentists (99.7% of the 2195 dentists surveyed) had adopted additional infection control measures in their practices [42]. Also, in March 2020, the use of telehealth in the US increased by 154% over the same period in 2019 [43]. In addition, the knowledge and attitude of dentists towards teledentistry changed. A report on 5370 dentists in Colombia showed that knowledge of teledentistry increased by 62.7% and its use by 42.5%. Additionally, 59.6% of dentists consider continuing to use teledentistry once the pandemic is over [44].

The surgical or invasive management of dental caries is still the treatment of choice worldwide [45], although a number of non-invasive and minimally invasive methods would allow caries to be treated more successfully [14], reducing pain and financial costs [46]. Evidence shows that research results take decades to be incorporated into routine clinical practice [47]. For dental sealants it has been a long time to be introduced in Latvia as this procedure is not paid for by public dentistry; thus just less than half of Latvian dentists are ready to use them for caries treatment. It is not clear to us why most Latvian dentists stated that they were unlikely to use a minimally invasive technique such as the Hall technique during the period of recommendation not to use or avoid AGPs for caries treatment in primary dentition. As Hall's technique is an effective non-AGP caries treatment and restorative technique [48], the only explanation we can think of is that Latvian dentists are unfamiliar with it. This suggests that there is an opportunity to disseminate this technique in Latvia. Finally, it should be noted that these results refer to dentists' statements about what they would do in the event of a national emergency and under two types of national recommendations about AGPs.

In Latvia, the public sector mainly provides paediatric dentistry, so policy decisions need to be focused on effective methods for children. Currently, the financing is mainly for restorative treatment of caries, pulp treatment, and extractions, all of which are available under both local and general anaesthesia. However, the media regularly report on the limited availability of dental care in emergencies. It is important to recognize that, according to scientific evidence, restorations do not reduce caries-related complications (pain and inflammation) or the occurrence of new carious lesions [49]. Latvian health policies should aim to be caries-free children, which is in line with the objective and sub-objectives of Action line No. 5 of the Public Health Guidelines [50]. To achieve these, changes in remuneration systems should be made, introducing a hybrid payment system which should include paying for the absence of diseases, bonus payments for high-risk patients or special needs patients, bonuses for evidence-based techniques, and bonuses for sound data collection [51]. The list of accepted medical technologies currently valid in Latvia also needs to be updated; it needs to be assessed which technologies are outdated and should no longer be used and which evidence-based methods are not included and should be incorporated as medical technologies. We also think that introducing evidence-based methods would decrease emergencies due to caries, making public service better available.

This study shares the limitations inherent to survey studies. Therefore, it should be borne in mind that the results refer to what dentists state they would do. However, even with this potential bias, it is possible to see a trend in the results towards the acceptability of non-invasive and fewer aerosol-generating methods for caries treatment. The response rate obtained is in line with what usually occurs with survey studies [52]. Thus, the online response rate was much lower (21.6%) than that obtained with paper questionnaires (56.2%). However, the total number of questionnaires obtained is sufficient to provide nationally representative results. It should be noted that the answers were in alternative formats and did not accept free text. However, the experts who participated in the development of the questionnaire agreed on the answers that captured the greatest variability of possible treatments in general. Another limitation is that although we did not detect that the specialty explained the change in treatment decision, we must consider that only 7% of the sample (29/373) declared themselves to be specialists. The form did not include details of which specialty.

In conclusion, Latvian dentists are willing to maintain patient care and treat caries during the pandemic and state that they prefer to use non- or minimally invasive and fewer aerosol-generating methods for the treatment of uncomplicated caries under recommendations to avoid AGPs.

## Supplementary Information

**Additional file 1**. Tabulated survey results.

## Data Availability

The data supporting this study's findings are openly available in the Riga Stradins University Dataverse at https://doi.org/10.48510/FK2/PZL9DW, and a xls spreadsheet with the raw data is included as Additional file 1.
